# Health Management Workforce Capacity-Building in Liberia, Post-Ebola

**DOI:** 10.5334/aogh.3250

**Published:** 2021-10-08

**Authors:** Kristina Talbert-Slagle, Freda Koomson, Neima Candy, Sean Donato, Jane Whitney, Chelsea Plyler, Nikole Allen, George Mourgkos, Regan H. Marsh, Lila Kerr, Rex Wong, Mosoka Fallah, Bernice Dahn

**Affiliations:** 1 Yale University, US; 2 National Public Health Institute of Liberia, University of Liberia College of Health Sciences, LR; 3 Brigham and Women’s Hospital, Partners In Health, US; 4 University of Global Health Equity, RW; 5 University of Liberia College of Health Sciences, LR

## Abstract

Following the Ebola crisis in Liberia in 2014–15, the Liberian Ministry of Health developed a strategy to build a fit-for-purpose health workforce, focusing on both health care providers and health managers. To help fulfill national capacity-building goals for health management, a team of faculty, staff, and practitioners from the Yale School of Medicine, the University of Liberia, the National Public Health Institute of Liberia, and the Ministry of Health collaboratively developed and launched the health management program in Liberia in July 2017. The team worked to build specific management and leadership competencies for healthcare workers serving in management and leadership roles in Liberia’s health sector using two concurrent strategies—1) implementation of a hospital-based partnership-mentorship model in the two largest hospitals in the capital city of Monrovia, and 2) establishment of an executive education-style advanced Certificate in Health Systems Leadership and Management at the University of Liberia. Here we describe the health management program in Liberia, its focus, and its evolution from program launch in 2017 to the present, as well as ongoing efforts to transition program activities to local partner ownership by the end of 2021.

## Introduction

Effective health management is necessary for ensuring quality, improving processes, and reaching target outcomes within any health system, but particularly where resources are scarce [1,2]. Recognizing the fundamental, cross-cutting importance of health management for health systems strengthening, programs to build health management capacity have been implemented in various resource-constrained settings globally [3,4]. These programs have routinely utilized a multi-faceted approach, focusing simultaneously on individual, facility, and system-level capacity-building, to strengthen management and improve health outcomes [5].

Health management capacity-building was a key feature of the West African country of Liberia’s post-Ebola Health Workforce Program strategy [6]. Health leaders in Liberia, who had already been working to rebuild their country’s health system after a 14-year period of civil war ended in 2003, faced devastating losses nationwide due to the Ebola Virus Disease (EVD) crisis in 2014–15 [7]. Liberia not only had the highest number of EVD deaths globally, with more than 4,800, but also lost a crippling 8% of its physicians, nurses, and midwives [8]. The Government of Liberia articulated a strategy for building a resilient health system post-Ebola that included a fit-for-purpose health workforce focused on five priority cadres: physicians, nurses, midwives, community health workers, and health managers. Stating that “Effective management of health facilities and staff is essential to the optimal delivery of training and services. The 2014–2015 EVD outbreak demonstrated the heightened importance of such management during times of public health crisis,” Liberia’s Health Workforce Program strategy emphasized the importance of building capacity in health management to help achieve the country’s overall goal of building a resilient health system [10].

The Resilient and Responsive Health Systems Initiative (RRHS), funded by the President’s Emergency Plan for AIDS Relief (PEPFAR) through the U.S. Human Resources and Services Administration (HRSA), supported implementation of the health management capacity-building component of the Liberia Health Workforce Program. Yale School of Medicine served as the lead implementing partner, accompanying the Liberian Ministry of Health, University of Liberia, and leadership of two hospitals in Monrovia: John F. Kennedy Medical Center (JFKMC) and Redemption Hospital (RDH). Here we describe the health management program in Liberia, its focus, and its evolution from program launch in 2017 to the present.

## Health Management Capacity-Building in Liberia, post-Ebola

A team of faculty, staff, and practitioners from the Yale School of Medicine, the University of Liberia, and the Ministry of Health collaboratively developed and launched the health management program in Liberia in July 2017. Informed by lessons learned and best practices from similar initiatives in Ethiopia, Liberia, Rwanda, and others [2,9,10], the health management program employed two concurrent strategies: 1) implementation of a hospital-based partnership-mentorship model in the two largest hospitals in the capital city of Monrovia, and 2) establishment of an executive education-style advanced Certificate in Health Systems Leadership and Management (CHSLM) at the University of Liberia (UL) to build management and leadership competencies in the health workforce.

## Implementing a Partnership-Mentorship Model in two major hospitals in Monrovia

The health management program in Liberia established a full-time, in-country health management team consisting of a Health Management Director and two Health Management Associates. The Director served as the lead for the program, collaborating closely with the Minister of Health and her designees, leadership of JFKMC and RDH, other RRHS partners, and health care practitioners in both hospitals to facilitate program implementation and ensure alignment with local priorities and goals. The two Health Management Associates were based full-time at JFKMC and RDH, where they collaborated with hospital administrators and supervisors in a shoulder-to-shoulder mentorship model to identify system and process gaps [6], engage in strategic problem solving, and develop and implement quality improvement (QI) projects. Implementation of QI projects has been shown to be an effective approach to facilitate real-time teaching and practice of health management skills and competencies to existing health managers [11,12]. During the first two years of the initiative, QI projects were focused on hospital-wide improvements and were developed in partnership with key managerial staff such as the Medical Records Supervisor, the Nursing Director, the Medical Director and/or the CEO. These QI projects were developed collaboratively with hospital staff and collectively accomplished the aim of building health managers’ skills in strategic problem solving and quality improvement, although with variable long-term impact. Illustrative QI projects are shown in ***Table 1***.

**Table 1:** Building health management capacity at JFKMC and RDH: Illustrative QI projects.


QI PROJECT	HOSPITAL	DESCRIPTION	GOAL

Revising Hospital Census Monthly Reporting	RDH	Created a database system for the nursing director to input departmental data, enabling automatic generation of pre-set monthly, quarterly, and yearly reports. Pilot time study of project impact indicated that processing time was reduced by 59%.	To reduce the process time in generating monthly, quarterly, and yearly reports

Computer literacy skills course for hospital staff	RDH	Provide ongoing computer skills training to department supervisors and staff in general computer literacy and Microsoft Word, Excel, PowerPoint, etc.	To improve administrative efficiency by utilizing computer technology in day-to-day work tasks.

Collaborative development and validation of 79 patient and hospital management Standard Operating Procedures (SOPs)	JFKMC	Development of 79 SOPs included work with the fiscal (financial), outpatient, and pharmacy departments. More than half of the SOPs focused on administrative and management processes. SOPs addressing outpatient department (OPD) flow included process mapping sessions with the JFKMC OPD Director and nursing staff.	Standardize and document patient, administrative, fiscal, and pharmacy management processes to train new staff and reduce patient wait times

Patient census database and staff database management mentoring	JFKMC	Developed patient census databases and mentored JFK Medical Records Department to more efficiently and accurately capture and report patient data for the Out-Patient, In-Patient, Pediatric and Emergency Departments.	Improve the accuracy and timeliness of patient census data analysis and reporting to inform decision-making


All of the projects described in ***Table 1*** were successful in building local managers’ capacity to utilize strategic problem solving and improve quality in response to specific organizational or administrative challenges identified by the local management team. Some projects did not continue in their original forms, however, due to issues such as staff turnover, technical challenges, shifting priorities, and changes in data management and reporting requirements from the Ministry of Health. Other projects, such as the computer skills classes at RDH and utilization of some Standard Operating Procedures (SOPs) at JFKMC, are still ongoing and are maintained by local health managers without foreign technical assistance.

In 2019, to align more closely with the goals of the funding organization (PEPFAR), the program pivoted to focus on strengthening management capacity within the two major infectious disease clinics in the capital city of Monrovia, based at both RDH and JFKMC hospitals. Although specific numbers of people living with HIV (PLHIV) who are or should be served by these clinics is difficult to determine, UNAIDS estimates that 43,000 adults (ages 15–49) were living with HIV in Liberia in 2019 [13]. Given that nearly 30% of Liberia’s population lives in and around the capital city of Monrovia [14], and that JFKMC and RDH have the two highest-volume HIV clinics in the country, the population of PLHIV needing and/or receiving care in these clinics is likely to be several thousand individuals, including those who are living with HIV but are undiagnosed. In late 2019, the infectious disease clinic at JFKMC, alone, had records for 2,459 patients.

After the health management program pivoted to focus on care and treatment for PLHIV in 2019, the team’s quality improvement efforts shifted toward specifically improving health outcomes in Liberia to meet the UNAIDS 90-90-90 goals, which set targets for 90% of all people living with HIV to know their status, 90% of people diagnosed with HIV to receive sustained antiretroviral therapy, and 90% of all people receiving antiretroviral therapy to have viral suppression [15]. Illustrative examples of QI projects implemented from 2019 to the present are shown in ***Table 2***.

**Table 2:** Building capacity in health management toward achieving 90-90-90 targets for PLHIV in Liberia: Illustrative QI projects.


QI PROJECT	HEALTH FACILITY/ORGANIZATION	DESCRIPTION	GOAL

Creation of CD4 and Viral Load database; mentorship of Infectious Disease Clinic staff on data management and analysis	Infectious Disease Clinic at JFKMC	Created database to capture and track patient CD4 and viral load results	Improve management of HIV patient records and identify viral suppression trends

Design and implementation of new Nursing Director’s Report for HIV clinic	Chest Clinic at RDH	Collaborative revision of a key-indicator clinic report submitted by supervisors to the Nursing Director on a monthly basis.	To provide a comprehensive overview of clinical outcomes ensuring that all key elements of 90-90-90 targets are included for efficient decision-making on resource allocation

Creation of job aid booklets for adult and pediatric HIV care and treatment, based on national guidelines	National AIDS, HIV, and STI Control Program	Pocket size ready reference booklets summarizing new 2020 Liberian HIV Guidelines supplied to every provider.	Improve accuracy and timing of service delivery, reduce errors in care of clients living with HIV and improve results relative to 90-90-90 targets

Ensure regular convening of partners; develop joint work and training plans	Infectious Disease Clinic at JFKMC; Chest Clinic at RDH	Monthly meetings with external partners, clinic supervisors, and institutional leadership at JFKMC and RDH for open discussion of all HIV-related projects and goals	Coordinate and streamline the work of RRHS and other partners and engage senior leadership for buy-in to improve service delivery, incorporate HIV clinic needs in hospital needs and priorities, ensure collective problem solving, and minimize duplication of efforts

Adaptation of pediatric dosing wheels to comply with national guidelines	National AIDS, HIV, and STI Control Program	Job Aid ready reference for dosing of pediatric HIV drugs supplied to every pediatric provider	Improve the accuracy and timing of care of infants exposed to and infected with HIV.

Design and development of a comprehensive Viral Load process	Infectious Disease Clinic at JFKMC	Viral load kit containing generic workflow, roles and responsibilities, job aids and accountability processes.	Improve Viral Load Suppression in clients living with HIV.


As with health management program QI projects that focused more broadly on building health and hospital management capacity prior to 2019 (***Table 1***), the QI projects represented by the examples in ***Table 2*** accomplished the overall program goals of building capacity of local managers to identify specific management-related problems, deploy strategic problem-solving strategies, and develop focused, feasible quality improvement projects in the infectious disease clinics at both JFKMC and RDH. For some specific projects, such as improving viral load testing to enable viral suppression for PLHIV, process improvements did not always yield consistent improvements in desired outcomes, such as increased viral load testing. Some of these challenges were due to system-level factors such as shortages of viral load cartridges, or local challenges such as intermittent electricity. For other QI projects, such as adaptation of pediatric dosing wheels and creation of job aid booklets to meet national guidelines, project outcomes led to sustainable tools and process improvements that remain in place. The overall effectiveness of the health management program approach, however, is illustrated by the fact that health management teams at both hospitals have now developed their own QI projects, along with plans to track progress, and are implementing those projects without foreign technical assistance. This indicates success in the overarching goal of the hospital-based partnership-mentorship model: to build the capacity of local health managers to identify management-related challenges, utilize strategic problem-solving strategies to develop solutions (often in the form of focused QI projects), and implement those projects, recognizing that sometimes the desired project outcome will not be achieved, but that the team will nonetheless learn and improve in health management skills and competencies for long-term, system-level impact.

## Certificate in Health Systems Leadership and Management, University of Liberia

In Liberia, health workers are frequently tasked with assuming managerial roles that require leadership and management competencies, and that confer considerable decision-making power over how resources—human, financial, and medical supply—should be allocated, without any formal management or leadership training. To address these challenges, the health management team from Yale University, the National Public Health Institute of Liberia, and the University of Liberia jointly designed an executive certificate program, the Certificate in Health Systems Leadership and Management (CHSLM). Building on curricula for similar programs implemented in other settings, the CHSLM was developed by all partners to fit the Liberian setting and needs of current health managers, with substantive input from Liberian colleagues and partners [16].

The CHSLM course includes ten in-classroom days divided into three modules over a nine-month period. Instructors utilize an experiential teaching method, including interactive lectures, facilitated discussions, team exercises, and ongoing mentorship, and participants complete team-based fieldwork focused on developing and implementing a quality improvement project in their workplaces. The course guides healthcare professionals through a strategic problem-solving process and professional skills’ enhancement experience through which they:

– Develop and apply key leadership and management skills,
– Address critical system problems with evidence-based strategies,
– Learn and practice effective management and accountable governance.

The in-service structure of the certificate program allows health care practitioners to participate in this educational opportunity with minimal disruption to their full-time work. Upon completion of the program, participants earn seven academic credits applicable to the UL Master of Public Health program if they wish to further their scholarship in public health. The course was approved by the University of Liberia’s leadership prior its launch in October 2018.

Two cohorts of CHSLM students have completed the program, and a third is underway. In follow-up self-assessments and a survey administered to graduates from the first cohort one year after completion of the program, respondents unanimously gave the program the highest score of 5 on a 1–5 scale when asked if the program enhanced their critical thinking, analytical, and problem-solving skills. As one respondent wrote, “investigative, critical thinking and analytical skills have improved my thought processes coupled with knowing how to set up various financial statements.” Another respondent described developing a standard operating procedure on bed-sore prevention & management, through which thirteen nursing staff were trained and the average monthly bed sore rate was reduced from 27.3% to 16.7%. ***Table 3*** provides a summary of CHSLM cohorts to date.

**Table 3:** Certificate in Health Systems Leadership and Management at the University of Liberia School of Public Health: Summary of three cohorts


COHORT	ACADEMIC TERMS	# PARTICIPANTS	HEALTH FACILITIES REPRESENTED (LOCATION)	DESCRIPTION

**1** (inaugural)	2018–2019	18	JFKMC (Monrovia) RDH (Monrovia)	Females: 14, Males: 4 1 deputy CEO (RN) 1 deputy CMO (MD) 2 hospital administrators 1 procurement director 1 compliance manager 1 nursing director (RN) 1 assistant nursing director (RN) 1 human resource director 1 medical record manager 1 nursing school administrator (RN) 7 nurse supervisors (RNs)

**2**	2019–2020	6*	JFKMC (Monrovia) RDH (Monrovia) JJ Dossen Hospital (Maryland County)** Liberia Medicines and Health Regulatory Authority (Monrovia)	Females: 5, Male: 1 1 hospital administrator (RN) 1 infectious disease clinic manager (HIV) (RN) 1 laboratory room supervisor (laboratory Technician) 2 HIV nursing supervisors (RNs)

**3**	2020–2021	16	Liberia National AIDS/HIV/STI control program (Monrovia) RDH (Monrovia) JFKMC (Monrovia) Bong Mines Hospital (Bong) New Sight Eye Clinic (Monrovia)	Females: 11, Males: 5 3 hospital administrators (1 MD) and 2 BSc in Administration 1 hospital consultant (MD) 2 HIV program supervisors (RN and PA) 2 Nurses training instructors (RN-Midwives) 1 laboratory room supervisor (lab technician) 7 nursing supervisors (RNs) supervising HIV units


JFKMC: John F Kennedy Memorial Medical Center RDH: Redemption Hospital CMO: Chief Medical officer CEO: Chief Executive officer RN: Registered Nurse MD: Medical Doctor PA: Physician Assistant *Due to COVID-19, web-based video call was utilized to accommodate distanced participation for a student who was in need of this consideration. **Maryland County is in the rural, underserved southeastern region of Liberia.

## Transfer of the Health Management Program to Liberian Ownership

Since the inception of the health management program in July 2017, a major goal has been gradual transfer of ownership and implementation responsibility for program activities, both health management capacity-building within the two major hospitals as well as the CHSLM at UL, to local partners and institutions. As the RRHS-funded health management program in Liberia begins its fifth and final year, this transfer process is well underway. In 2021, the health management team composition has shifted, and local hospital employees, many of whom have participated in QI projects and the CHSLM program over the past four years, have joined the team as permanent, local health management mentors. These local team members will continue to build local health management capacity through the partnership-mentorship approach and establish a network of professional health managers in Liberia.

For the CHSLM, transfer of ownership began with the inaugural cohort, when Yale faculty co-taught with faculty from UL. As the CHSLM program has progressed, Yale faculty have shifted from leadership to supporting roles with their UL counterparts in classroom instruction, student mentorship, and administrative processes required for implementation of the CHSLM program. Full handover of the program to the UL College of Health Sciences was completed in January 2021.

## Reflections and Lessons Learned

Continuous engagement and collaborative involvement of institutional leadership at both John F. Kennedy Medical Center and Redemption hospital, the University of Liberia, and the National AIDS Control Program, as well as with the Minister of Health, the President and senior leadership of the University of Liberia, and several others, have been critical to the success of the health management program. Implementation of key health management program deliverables would not have been possible without the hard work, dedication, and amenability to change exhibited by local partners.

As the RRHS program shifted in 2019 from a broad, systems-based emphasis to a focus on meeting 90-90-90 targets for PLHIV in Liberia, the health management partnership engaged in many adjustments to find ways to optimize building health management capacity within the HIV-focused framework. The COVID-19 pandemic also introduced challenges to program implementation, causing a shift to remote work for all health management team members and many of their partners in 2020 [17]. Both of these program shifts, although significant and challenging, were eased by strong, responsive consortium leadership and flexibility and creativity from all partners.

As in many multi-year international programs, turnover among staff and leadership at multiple affiliated institutions and agencies, including national changes following the 2018 presidential election in Liberia, necessitated building new relationships and institutional memory, which was often challenging. Ongoing team meetings, thorough documentation of program activities, workplans, and expected shifts—including transition planning documents—and frequent reporting reduced confusion and minimized redundancies that could otherwise have arisen due to shifts in personnel.

The health management program in Liberia built upon strong foundations established in Liberia and other countries by partners known to the consortium from the outset. The Yale Global Health Leadership Institute had previously partnered with Mother Patern College of Health Sciences to establish a similar health management certificate program around 2008, and many past Liberian participants in that and other Yale health management programs consequently became local, high-level champions of health management capacity-building in Liberia. In addition, publications and other public scholarship by health management practitioners worldwide who had implemented similar programs in other resource-constrained settings established best practices (and “what not to do”) that helped inform the design and implementation of the health management program [9,12,13,14,15,16, 23].

## Conclusion

The health management program in Liberia was designed to build health management capacity, following the vision and strategy of the Ministry of Health and the Liberia Health Workforce Program, using a two-pronged approach: hospital-based partnership-mentorship at two major hospitals in the capital city, and implementation of a permanent executive education program at the University of Liberia to build capacity for existing health managers in Liberia’s health systems. All components of the program were designed to enable transfer of health management capacity-building activities to local ownership within the five-year RRHS program period, and the program is on track to meet these goals.

Health management competencies are critical both to enable health systems strengthening broadly and to meet targeted health outcomes in vertical disease programs. Investments in health management capacity-building have helped maximize efficient use of resources (including clinician time, equipment and commodities, and physical space) to benefit patients and improve health outcomes. As mentees and CHSLM graduates progress in their careers, their exposure to and experience applying proven leadership and management skills and competencies, such as strategic problem-solving, using evidence for decision-making, applying management competencies to daily practice, etc. will continue paying dividends, both as they face new challenges and mentor others to do the same. The health management program in Liberia can serve as a model for other efforts that seek to build health management capacity in resource-limited settings and enable local higher education institutions to offer locally-appropriate, tailored educational programs to strengthen health management system-wide.
